# Demographic buffering and compensatory recruitment promotes the persistence of disease in a wildlife population

**DOI:** 10.1111/ele.12578

**Published:** 2016-02-11

**Authors:** Jenni L. McDonald, Trevor Bailey, Richard J. Delahay, Robbie A. McDonald, Graham C. Smith, Dave J. Hodgson

**Affiliations:** ^1^ Centre for Ecology and ConservationCollege of Life and Environmental SciencesUniversity of ExeterPenrynCornwallTR10 9FEUK; ^2^ College of Engineering, Mathematics and Physical SciencesUniversity of ExeterExeterDevonEX4 4QFUK; ^3^ National Wildlife Management CentreAnimal and Plant Health AgencyWoodchester ParkGloucestershireGL10 3UJUK; ^4^ Environment and Sustainability InstituteCollege of Life and Environmental SciencesUniversity of ExeterPenrynCornwallTR10 9FEUK

**Keywords:** Badger, Bayesian, bovine tuberculosis, demographic buffering, demography, density‐dependence, Integrated Population Model, life history, *Meles meles*, wildlife reservoir

## Abstract

Demographic buffering allows populations to persist by compensating for fluctuations in vital rates, including disease‐induced mortality. Using long‐term data on a badger (*Meles meles* Linnaeus, 1758) population naturally infected with *Mycobacterium bovis*, we built an integrated population model to quantify impacts of disease, density and environmental drivers on survival and recruitment. Badgers exhibit a slow life‐history strategy, having high rates of adult survival with low variance, and low but variable rates of recruitment. Recruitment exhibited strong negative density‐dependence, but was not influenced by disease, while adult survival was density independent but declined with increasing prevalence of diseased individuals. Given that reproductive success is not depressed by disease prevalence, density‐dependent recruitment of cubs is likely to compensate for disease‐induced mortality. This combination of slow life history and compensatory recruitment promotes the persistence of a naturally infected badger population and helps to explain the badger's role as a persistent reservoir of *M. bovis*.

## Introduction

Wildlife hosts are important contributors to endemic and emerging infections worldwide, posing threats to human and livestock health (Gortázar *et al*. 2007). The importance of wildlife hosts is heightened when they sustain chronic infections, creating persistent reservoirs of disease. Understanding the processes favouring the long‐term persistence of populations of infectious hosts is essential for predicting the dynamics of wildlife diseases, and their management. However, simple censuses of wildlife host abundance and disease prevalence generally fail to provide a mechanistic understanding of host demography and life history, thus limiting the effective management of disease risks. Our goal is to determine whether the life history and demography of the European badger (*Meles meles*) predisposes it to being a reservoir of *Mycobacterium bovis*.

Density‐dependent compensation is a key mechanism that promotes the persistence of wildlife populations harbouring fatal diseases (Anderson & May 1981). If local abundance is reduced through disease‐induced mortality, the remaining healthy individuals could respond to reduced intraspecific competition by increasing recruitment and/or survival, thus offsetting losses. Theory suggests that the methods of compensation that promote persistence of wild reservoir populations are linked inextricably to their life‐history strategy. Specifically, the demographic buffering hypothesis (Gaillard & Yoccoz 2003), a corollary of the slow‐fast life‐history continuum (Promislow & Harvey 1990; Pfister 1998), states that natural selection will favour the buffering of important vital rates against temporal environmental variation. From this theory, we can suggest two interlinked predictions. The first prediction is that slow living adult mammals should buffer their survival against fluctuations in external and internal pressures, including density, but then experience fluctuations in reproductive effort (Gaillard *et al*. 1998, 2000), while fast‐living species should buffer their reproduction and experience fluctuations in rates of survival (Korpimaki *et al*. 2004). The second prediction is that density‐dependent recruitment is likely to be more effective than density‐dependent survival as a mechanism to compensate against losses due to disease, and to be more prevalent among slow life‐history strategists compared to those with a fast life history.

In theoretical studies, density‐dependent recruitment has been highlighted as a key population stabiliser for disease systems under frequency‐dependent (Thrall *et al*. 1993) and density‐dependent (Anderson & May 1981) transmission. Density‐dependent recruitment is proposed to compensate for the loss of infected individuals in wild populations that under normal circumstances exhibit demographic characteristics of a slow life‐history strategist (low recruitment and high adult survival), resulting in the persistence of wild reservoirs of disease (Arthur *et al*. 2004; Muths *et al*. 2011; Tobler *et al*. 2012). Conversely, relatively fast life histories, for example of the Tasmanian devil (*Sarcophilus harrisii*), predispose species to maximising both litter size and the proportion of females breeding under normal conditions, therefore limiting the scope for further increases in reproduction to compensate for population declines due to disease epidemics (Lachish *et al*. 2009). The alternative mechanism of compensation is density‐dependent survival among individuals not lost to disease: this might be feasible for species with fast life histories, but not for those with slow life histories that have high and stable survival with limited capacity for increase at low density (Gaillard *et al*. 1998).

To explore the theory that population persistence in the face of a chronic disease fits with a species' position on the slow‐fast life‐history continuum, we analyse the demography of a high density infected badger population (Delahay *et al*. 2013). Badgers have the characteristics of a slow life‐history strategist and are an important host of *M. bovis*, a source of infection to cattle (Donnelly *et al*. 2006) in the UK and Ireland, providing a topical and important study system. Several lines of evidence offer conflicting predictions regarding the importance of disease processes for fluctuations in badger abundance. Badgers with advanced tuberculosis, determined by positive culture of *M. bovis* and hence indicative of an infectious state, experience significant weight loss (Tomlinson *et al*. 2013) and high mortality (Graham *et al*. 2013). Hence, *M. bovis* infection might drive fluctuations in rates of survival. However, *M. bovis* prevalence does not appear to affect population dynamics (Delahay *et al*. 2013). It is possible that density‐dependent recruitment is a stabiliser of this population. In any given year, an estimated 29% (15–50%) of females breed (Carpenter *et al*. 2005), however, reproductive suppression is likely to occur in badgers of poor social status and/or body condition at high densities due to increased female‐female competition (Woodroffe & Macdonald 1995) and reduced resource availability. Yet most research on density‐dependence in badgers has focused on the impact of intraspecific competition on body mass of adults and, despite links to fecundity (Cresswell *et al*. 1992; Macdonald *et al*. 2002), no assessment of compensatory density‐dependence has been made.

Here, we exploit advances in Bayesian integrated population modelling (Besbeas *et al*. 2002) to provide a unified assessment of badger population dynamics. Demographic processes are often inferred from capture mark recapture (CMR) data using a range of models (Lachish *et al*. 2007; Muths *et al*. 2011; Graham *et al*. 2013). However, integrated population models (IPMs) combine models that analyse census and CMR data. This method provides a fully mechanistic framework to simultaneously estimate population growth, survival, and recruitment rates, and regress these against (potentially hierarchical) covariates.

We examine whether properties of badger life histories predispose infected populations to persist. Using long‐term CMR data from a population of badgers naturally infected with *M. bovis*, we ask: (1) Where does the badger's life history sit on the slow‐fast continuum? (2) What is the impact and relative importance of the prevalence of diseased individuals and host density on badger vital rates? (3) To what extent does badger life‐history buffer population dynamics against fluctuations in disease prevalence? Overall, we found density‐dependent recruitment to be a strong driver of badger population dynamics. This demographic mechanism underpins the persistence of populations of this slow living species despite the prevalence of a fatal disease.

## Materials and Methods

### Study site and data characteristics

Woodchester Park in Gloucestershire, UK is the site of a long‐term CMR study of a high density badger population naturally infected with *M. bovis* (Delahay *et al*. 2013). The study area comprises *c*. 7 km^2^ of mixed woodland, grassland and arable. Each badger is given a unique identifying tattoo when it is first caught and at every capture event the location of capture, sex, age class (cub or adult) and *M. bovis* infection status using a range of diagnostic tests was recorded (for detailed methods see Delahay *et al*. (2000)). Data for the present study originated from trapping records for 20 core study groups. The high‐density badger population at Woodchester Park is typified by a low level of movement of individuals between social groups, with immigration and emigration found to be minimal in previous studies (Rogers *et al*. 1997, 1998). Consequently, our analysis assumes a closed population.

From this information, two data sets were constructed. First, annual capture histories, spanning from 1984 to 2005, were created for each individual badger living in 20 social groups. In total, 1521 individuals were included in this CMR data set, comprising 816 females and 705 males, of which 700 and 613, respectively, were first caught as cubs. Thus, the CMR data set differentiated between individuals marked as cubs and individuals marked as adults, and those that were male and female. Second, we recorded the annual number of unique badgers aged 1 year or older that were caught to provide an annual pre‐breeding survey data set. We note that, although a high proportion of badgers (0.78 females; 0.82 males; Table S1) are caught annually, the survey provides an index, not absolute measure, of the number of badgers in the population.

### Bayesian integrated population model

An integrated population model (Besbeas *et al*. 2002) was built in a Bayesian framework to analyse the CMR and survey data sets simultaneously. Specifically, CMR data and survey data were combined into a single framework to estimate between‐year survival, annual recruitment rates and population change. The core of the IPM is a population projection model, which maps various demographic rates for different age classes. We have previously demonstrated sex differences in badger mortality rates, with male mortality exceeding that of females (Graham *et al*. 2013), and so we developed a two‐sex, two‐age (cub and adult) structured population projection matrix with an annual time step. We calculated a *per capita* annual fecundity rate across the population of all badgers aged 1 year and older, thereby estimating a parameter analogous to recruitment rates that can be compared directly to survival in terms of its contribution to population growth. The birth of cubs is strictly seasonal in badgers, most likely occurring in February (Roper 2010). Consequently, CMR data cannot tell us about mortality of offspring prior to their emergence from natal setts in the spring and therefore the fecundity rate (termed recruitment) used throughout this paper is an integrated measure of reproduction and early cub survival.

### Estimating covariates: density and disease prevalence

Yearly estimates of density and disease prevalence were calculated to be used as covariates in the IPM. Density estimates were derived from the POPAN formulation (Schwarz & Arnason 1996) of Jolly‐Seber models in the program MARK (White & Burnham 1999). This model accounts for the yearly variation in recapture rates to provide yearly population abundance estimates. Models were fitted using the log link function for population size and tested and adjusted for overdispersion using the program RELEASE implemented from program MARK.

The prevalence of disease due to *M. bovis* was inferred from the number of infectious badgers, disclosed by positive bacterial culture from clinical samples of faeces, urine, sputum and swabs of abscesses and/or bite wounds (Gallagher & Horwill 1977). The culture test has low sensitivity for detecting infection *per se*, but is a useful and highly specific indicator of animals with advanced and infectious disease because it detects active excretion of live bacilli (Drewe *et al*. 2010). Yearly estimates of the number of infectious badgers were calculated by accounting for temporal variation in recapture probability using the POPAN modelling approach described above. These were then divided by the annual abundance estimates to provide a yearly index of the proportion of diseased, hence infectious, animals in the total population. Both density and disease prevalence estimates were incorporated as explanatory covariates within the IPM.

We tested assumptions regarding differential survival between age classes (cubs and adults) and between sexes, and whether covariates affected these cohorts equally (i.e. have additive effects) or differently (i.e. have interaction effects). We evaluated the best model structure using deviance information criterion values (DIC) (Spiegelhalter *et al*. 2002). We found most support for sex‐specific survival parameters but less support for incorporating differential cub and adult survival (ΔDIC > 23), a result that is in agreement with an earlier analysis of survival (Graham *et al*. 2013). We focus on sex‐specific models in our results. Disease and density models were built first and, to ensure our results were robust to the influence of environmental variables, weather covariates were incorporated (see Supporting Information 1.1).

### Components of the IPM

Using the IPM framework we modelled the log of recruitment (*f*) parameters and the logit of survival (ϕ) parameters as linear functions of covariates (*x*; disease prevalence, population density and weather) using the following linear relationships:

$$\begin{array}{cc} \text{logit}\phi_{t}= & \alpha +\sum_{j=1}^{n}\beta_{j}x_{j,t}+\varepsilon_{t}\\ \text{log}f_{t}= & \alpha +\sum_{j=1}^{n}\beta_{j}x_{j,t}+\varepsilon_{t},\\ \varepsilon ∼ & N(0,\sigma^{2}) \end{array}$$

where *x*_*j*,* t*_ are the values of the standardised *j*th covariate over time *t*, βs are the regression coefficients for each covariate and ɛ is the residual temporal variation providing estimates of unexplained variance ($\sigma_{Residual}^{2}$). We also fitted a model without covariate effects to gain an estimate of total temporal variance ($\sigma_{Total}^{2}$). The proportion of variance explained by the covariate effects is then estimated as $\left(\sigma_{Total}^{2}-\sigma_{Residual}^{2}\right)/\sigma_{Total}^{2}$ (Kéry & Schaub 2012). To assess the credibility of covariate effects we calculated the probability that their effects were positive [*P*(β > 0)] or negative [*P*(β < 0)], and to emphasise the relative strength of regression coefficients, covariates were standardised to have a mean of 0 and standard deviation of 1.

We adopt a Bayesian implementation of the IPM using vague normal N(0,10^4^) prior distributions truncated to lie in the interval (−5,5) for the unknown regression parameters (β), vague prior distributions for the log of recruitment N(0,10^4^) and vague prior distributions for the variance parameters on the standard deviation scale U(0,10). Informative uniform prior distributions spanning survival rates found previously (Graham *et al*. 2013) were set to achieve faster convergence for mean survival U(0.4,0.95).

### The joint likelihood

An IPM constructs likelihoods for the two separate data sets, composed of a state‐space model for the survey data and a CJS model for the capture histories (Kéry & Schaub 2012). The state‐space model is determined by a state and observation process. The state process describes the true but unknown population trajectory under the population model. This component accounts for demographic stochasticity by modelling the number of 1 year old badgers using a Poisson distribution, and the number of adults using a binomial distribution. The observation process accounts for observation error, which links the observed survey data (y) to the population size (N) with a Poisson distribution. The likelihood of the state space model is a product of the observation and process equations. This likelihood estimates total population size, survival of each age class (a), recruitment and observation error ($N,\Phi_{a},f,\sigma_{y}^{2}$ respectively). The CMR data (m) were analysed via a CJS model, which estimates year‐specific survival rates. The frequency of individual capture histories, which uses m‐array formulations, follows a multinomial distribution. This likelihood includes data for those badgers first caught as adults and cubs of both sexes, thus estimating survival parameters for both sexes (s) and age classes (Φ_s,a_) in addition to recapture probability (*p*). Combining these likelihoods formulates the joint likelihood of the IPM.

$$L_{IPM}\left(y,m|N,\phi_{a,s},f,p,\sigma_{y}^{2}\right)=L_{SS}\left(y,|N,\phi_{a},f,p,\sigma_{y}^{2}\right)\times L_{CJS}\left(m|\phi_{a,s},p\right)$$

### Model fitting and assumptions

Models were specified within R version 3.1.1 (R Development Core Team 2013), using the package R2WinBUGS version 2.1‐21 (Sturtz *et al*. 2005) to call WinBUGS 1.4 (Lunn *et al*. 2000), within which the models were run, and from which results exported back to R. Convergence of the chains was assessed by visually checking mixing of the chains and more formally using the Brooks–Gelman–Rubin criterion ($\hat{r}$ (Brooks & Gelman 1998)). Initial trials with three independent chains found that convergence ($\hat{r}\;<\;1.02$) was reached after 3000 iterations. Following the initial trial we ran three chains of 10 000 with a burn‐in of 3000 for each analysis and retained every 10th value, yielding a sample size of 2100 iterations. There is no established goodness of fit test for integrated population models, but when we tested the fit of the CMR model component in program MARK, the data were not overdispersed ($\hat{C}\;<\;1.9$).

## Results

**Figure 1:**
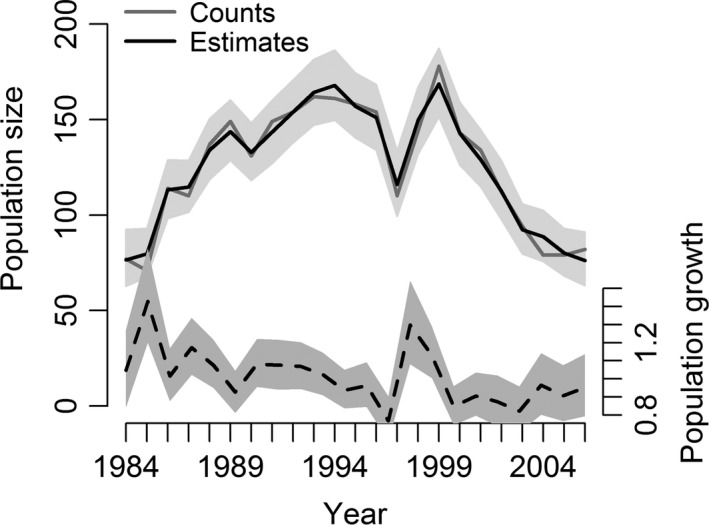
Observed counts and estimated population size, alongside between‐year population growth rates (dashed line), of the Woodchester Park badger population. Shaded regions represent 95% credible intervals (CRI) of estimates.

The mean population growth rate of the Woodchester Park badger population between 1984 and 2005 was 1.00 (95% credible interval, CRI = 0.99–1.01), but this apparent stability masks fluctuations in yearly growth rates between 0.78 and 1.40 (Fig. 1). Female badgers had a higher annual survival rate than males (mean female survival = 0.74, 95% CRI = 0.71–0.78; mean male survival = 0.68, 95% CRI = 0.64–0.72). Mean annual *per capita* recruitment was 0.41 (95% CRI = 0.34–0.48).

### The effect of disease and density

In the following text we refer to IPM results in which we explore the influence of the previous year's disease prevalence (D), and of the previous year's badger density (N) on both survival (S) and recruitment (R).

**Figure 2:**
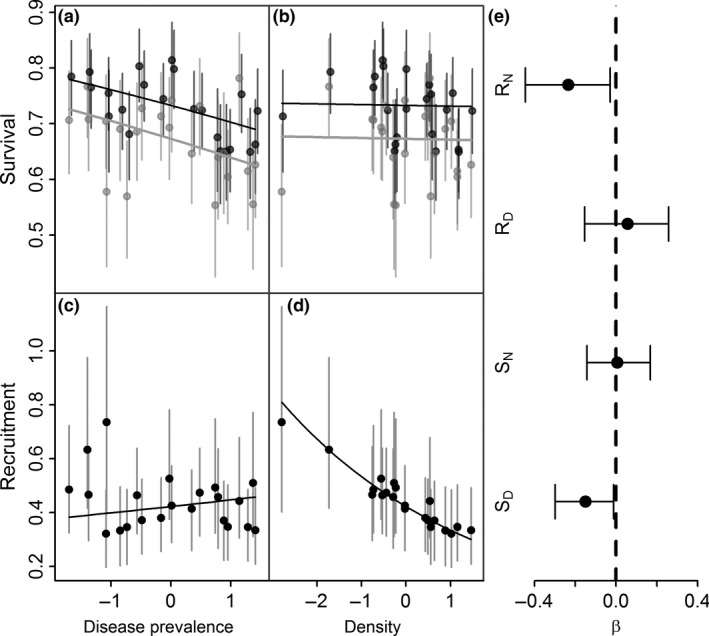
The influence of standardised density_t−1_ and disease_t−1_ on (a–b) survival of male (dark grey) and female (light grey) badgers and (c–d) recruitment rates in a badger population, showing the predicted relationship and the corresponding posterior means (points) and 95% CRI (vertical bars) from an IPM along with the predicted line from the posterior regression coefficients. (e) Regression slopes (β) describing the relationship between demographic rates; recruitment (R) and survival (S), and covariate effects; disease (D) and density (N). The posterior mean is displayed alongside the corresponding 95% credible intervals.

Density‐dependent regulation appears to act mainly via recruitment, not survival (credibility for recruitment = 0.99; for survival = 0.27). Conversely, the credibility of negative impacts of disease prevalence was low for recruitment (0.43) but high for survival (0.98). After standardising the covariates to have zero mean and a standard deviation of one, density and disease were found to have contrasting effects on survival and recruitment. Survival declined with increasing disease prevalence (posterior mean slope ${\hat{\beta }}_{D}$ = −0.15, Fig. 2) but was not affected by density (${\hat{\beta }}_{N}$ = 0.01, Fig. 2), whereas recruitment declined with increasing density (${\hat{\beta }}_{N}$ = −0.24, Fig. 2) but not with disease prevalence (${\hat{\beta }}_{D}$ = 0.06, Fig. 2). Dropping uninformative effects of density on survival and of disease prevalence on recruitment was favoured by DIC selection (ΔDIC > 8) and increased the strength and credibility of the informative covariates. Additionally, the inclusion of interactions between disease prevalence and badger sex was not well supported by DIC values (ΔDIC > 8) and did not result in any substantial change in the slope parameters.

### Components of variation in recruitment and survival

Our IPM (S_D_, R_N_) explained 42% of the variance in annual recruitment rates. Male and female badgers had similar levels of temporal variation in survival, but they differed in terms of the relative contributions of disease variables. Disease prevalence explained 3% of among‐year variance in males but 30% in females.

Our results above were robust to inclusion of weather covariates (full details in Supporting Information). The only notable difference was a change in the impact of disease on survival (${\hat{\beta }}_{D}$ changed from −0.15 to −0.03) due to an interactive effect with autumn temperature (${\hat{\beta }}_{D\times AT}$ = −0.13, posterior probability of negative slope = 0.92). This result highlights the effect of disease is dependent on autumn temperature (Fig. S1). Even with the inclusion of weather drivers, recruitment remained strongly density‐dependent (${\hat{\beta }}_{N}$ = −0.24, posterior probability = 0.98, Fig. S2). Full details of weather effects are in the Supporting Information.

### Correlations

Recruitment correlated most strongly, and positively, with population growth rate (r = 0.78). Survival rates for males and females were also positively correlated with population growth rate, but to a lesser extent (r = 0.54 and 0.64 respectively). The coefficient of variation (CV) was calculated for all demographic rates and revealed little year to year variation in female (CV = 7.74%) or male survival (CV = 9.76%) compared to recruitment (CV = 34.62%).

## Discussion

We have described the principal mechanisms governing variation in vital rates and their relative impact on the dynamics of a population of wild mammals carrying an economically important, infectious, zoonotic disease, using an integrated population model. Resource limitation prevents organisms from simultaneously maximising their survival and reproductive output. Thus, badgers exhibit a pattern of demographic variability (high but stable survival vs. low but variable recruitment) similar to that observed in other slow living mammals (Gaillard *et al*. 2000). Numerous ecological pressures are associated with life at high density, including competition for resources and increased frequency of aggressive encounters (Cresswell *et al*. 1992; Jenkins *et al*. 2012). Given the large influence of survival on fitness, the insensitivity of individual survival to exogenous fluctuations is advantageous (Pfister 1998; Gaillard *et al*. 2000), increasing individual fitness and hence promoting population stability (Saether *et al*. 2013). In addition, we reveal that density‐dependent recruitment, a key trait of this slow life‐history strategist, compensates population dynamics for losses due to disease‐induced mortality.

Our study identifies badgers as an effective reservoir of *M. bovis*, having relatively stable population dynamics, coupled with permanent maintenance of *M. bovis* within the population, despite widespread infection and disease‐induced mortality. In this system, host recruitment seems robust to disease prevalence, supporting the continued birth of susceptible cubs. Given that *M. bovis* infection does not impact female reproductive activity or success (Tomlinson *et al*. 2013), any loss of diseased individuals will be compensated for by density‐dependent recruitment and increased reproductive success. This result agrees with the theory that links disease persistence with demographic strategy (Anderson & May 1981; Thrall *et al*. 1993).

Our findings have implications for management of bovine tuberculosis in badgers, as density‐dependent recruitment will influence the extent and rate of recovery of populations that are subject to human management for the control of disease. Management options include culling, fertility control and vaccination. Compensatory density‐dependent recruitment in response to culling will increase the supply of susceptible badgers and could increase the number of infectious individuals along with disease prevalence (Choisy & Rohani 2006). Fertility control might prevent the observed compensation for disease‐induced mortality, but its impact on disease prevalence is unclear. Management of disease rather than host, for example using vaccination, should reduce the loss of individuals due to disease‐induced mortality: our models suggest this would help to maintain high population density and therefore prevent any increase in recruitment.

Previous research into the influence of the slow‐fast life‐history continuum on reservoir traits has focussed on the susceptibility and infectiousness of hosts. Fast‐living species have been found to be more susceptible and have greater pathology due to reduced investment in immune defences for both micro‐ and macro‐parasitic infections (Johnson *et al*. 2012; Ostfeld *et al*. 2014). In this study, we address the demographic response to disease as a measure of reservoir persistence. Investment into both demographic and immunological mechanisms will be interlinked. We hope that this examination of demographic mechanisms will motivate future efforts integrating immunological and demographic responses to disease.

The ability of populations to persist in the face of disease is not limited to badgers. However, we are largely unaware of the demographic mechanisms underpinning the persistence of populations. A general pattern is emerging in the literature, revealing that fast‐living species are limited in their scope for reproductive compensation (Lachish *et al*. 2009) while long‐lived, or ‘survivor’, species can increase their reproduction in the face of population depression by disease (Muths *et al*. 2011) or other factors. This has been explored in aquatic ecosystems where density‐dependence plays a key role in the life‐history characteristics and population ecology of slow life‐history strategists (Goodwin *et al*. 2006) and a similar compensatory effect has been observed in some bird populations (Paradis *et al*. 2002). However, there is evidence that such compensatory effects will be ineffective against rapid increases in mortality, with low clutch sizes in birds (a trait of a slow life‐history strategist) associated with increased extinction risk (Bennett & Owens 1997). We suggest that a combination of chronic disease and density‐dependent recruitment will result in similar patterns of persistence across slow life‐history strategists. Research into other chronically infected wild animal populations of both fast and slow life histories, and across a wider range of pathogen life histories, will lend support to our understanding of these demographic processes.

The challenges involved in measuring population dynamics from longitudinal studies of animals in the wild are numerous, often requiring various single‐process analyses to obtain all desired parameter estimates. In the present study, we developed an integrated population model in a Bayesian framework, which provided increased flexibility and the capacity to look beyond temporal stochasticity and observation error to reveal demographic patterns in a wild badger population. This has particular applied value in helping to inform approaches for the management of *M. bovis* transmission amongst badgers and cattle, but we also recommend the development and use of IPMs to benefit our understanding of demographic processes in other wildlife systems. Using this framework, our study provides empirical evidence of how density‐dependent recruitment, a corollary of a slow life‐history strategy, can promote population persistence and the coexistence of disease and hosts in a wild population.

## Authorship

Research was conceived by DJH, GCS, RJD, RAM and JLM. Analyses were performed by JLM with advice from DJH and TB. Results interpreted by JLM, DJH and RAM. The manuscript was written by JLM and DJH with advice from ALL.
