# Supplementary material for: Demographic buffering and compensatory recruitment promotes the persistence of disease in a wildlife population
Source: Ecol Lett. 2016 Feb 11;19(4):443–9. doi: 10.1111/ele.12578 (PMC4790914; doi:10.1111/ele.12578)
Supplement: Supplementary file 1 [file ELE-19-443-s001.docx]

**Supplementary Material for McDonald et al.**

**1.1 Weather covariates**

We accounted for possible confounding factors in our analysis by considering weather processes. Weather data were obtained from 5 x 5 km gridded observation datasets provided by the Met office (www.metoffice.gov.uk/climatechange/science/monitoring/ukcp09/available/). A priori hypotheses were made based on previous work. Autumn and spring are key seasons related to periods of high energy investment amongst badgers, and weather conditions at these times have been shown to affect badger survival (Macdonald et al. 2010). We also considered weather during winter, with mild conditions previously associated with increased mortality, likely mediated by increased activity levels and exposure to road traffic accidents (Macdonald et al. 2010). We explored the impact of weather variables from spring (March-May), autumn (September – November) and winter (December-February) on survival and recruitment rates. Both seasonal and monthly variables were tested. Mean temperature and summed precipitation are key weather features (Macdonald et al. 2010) and were tested for the time intervals of interest. Additionally, the number of days of ground frost was included, which we hypothesised may influence badger demographic rates because frozen ground will compromise feeding by digging for invertebrates and plant material.

As inclusion of weather components exponentially increased the number of competing models and computation time, we conducted model selection of weather covariates in program MARK. Specifically, we used CJS models for survival estimates and Pradel models for recruitment rates (Pradel 1996). Weather covariates present in the ‘best’ candidate model set, as indicated by a MARK analysis, were: the impact of autumn temperature (AT) and autumn rainfall (AR) on inter-annual badger survival rates, and the impact of frost-days in January (FJ) and spring temperatures (ST) on concurrent recruitment rates.

**1.2 Results**

**Survival**

Autumn temperature had a strong influence on survival (β_AT_ = -0.169, Fig. S1) with a high posterior probability that surviving overwinter declined with increasing temperature in the preceding autumn (99% of slopes were negative). Inclusion of this temperature effect reduced the impact of disease prevalence on survival (β_D_ changed from -0.149 to -0.038), and the posterior probability of negative disease-dependence reduced from 0.98 to 0.71 (Fig. S1). This may be explained by the interactive effect of autumn temperature and disease prevalence (β_D*AT_ = -0.131, posterior probability of negative slope = 0.91), whereby under average autumn conditions, disease prevalence is limited in its effect on survival, but with increased autumn temperatures the negative effect of disease prevalence on survival rates also increases (Fig. S1). Autumn rainfall had a relatively weak negative impact on survival (β_AR_ = -0.067, posterior probability of negative slope = 0.86; Fig S1). Disease and weather covariates explained 33% of between-year variance in male survival and 41% in female survival.

**Recruitment**

Rates of recruitment increased with increasing numbers of January frost days (β_FJ_ = 0.218, posterior probability 0.95; Fig. S2). Recruitment increased with warmer spring temperatures (β_ST_ = 0.199, Fig. S2) but the posterior probability of this effect was 0.94.

In models that ignored density-dependent recruitment, spring temperature effects on recruitment were not observably different from zero. Density and weather explained 46% of variation in recruitment rates.


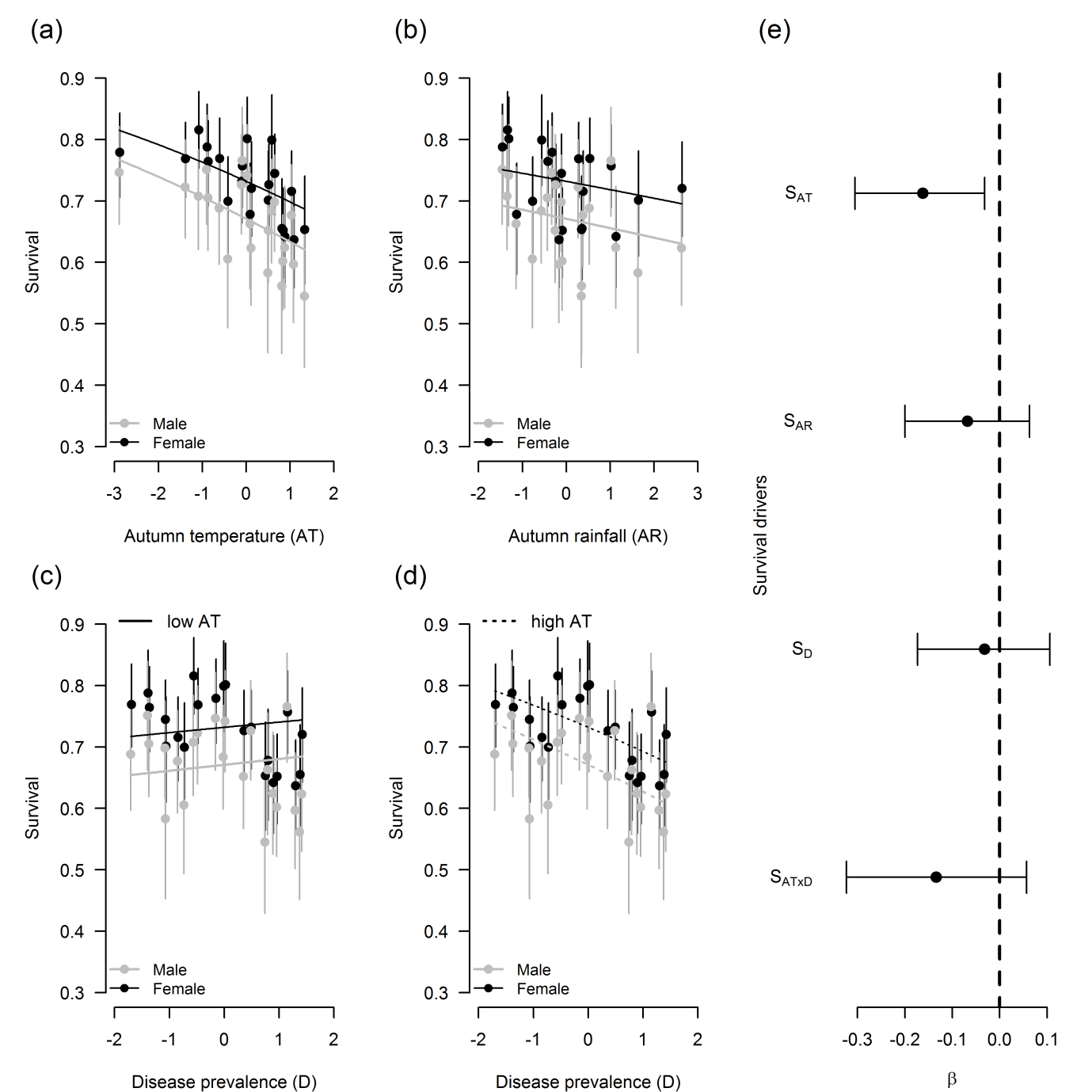


Figure S1. The effect of standardised covariates on survival rates as predicted by the IPM including (a-d); autumn temperature _t_ (AT), autumn rainfall _t_ (AR) and disease _t-1_ (D). The effect of disease prevalence on survival is weak under low autumn temperatures (c) but intensified at high autumn temperatures (d) due to an interactive effect between disease and autumn temperature (AT x D). (e) Regression slopes (β) describe the relationship between survival (S) and covariate effects. The posterior means are displayed alongside 95% credible intervals, on a logit scale.

**
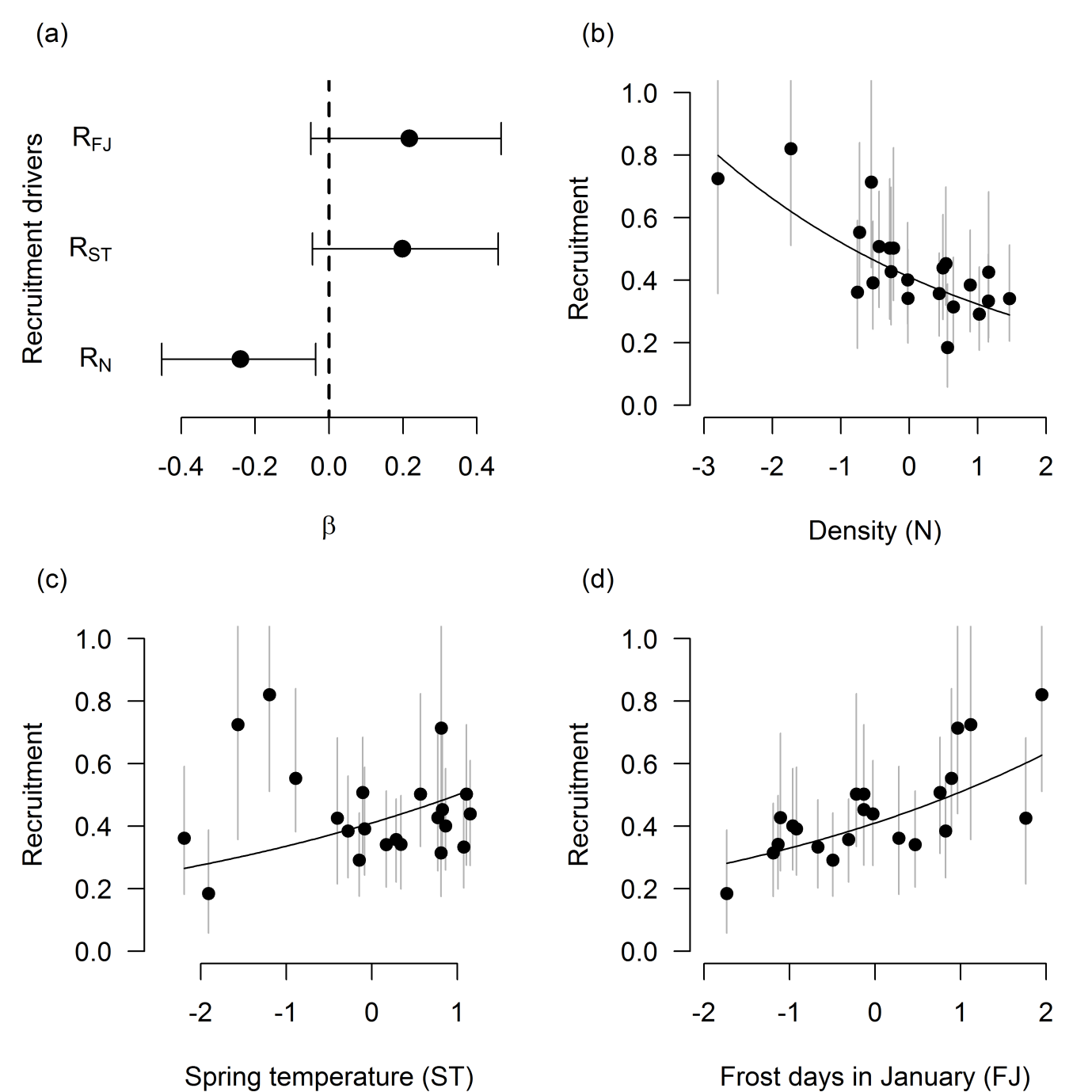
**

Figure S2. Regression slopes describing the relationship between recruitment (R) and covariate effects (a); density _t-1_ (N), frost days in January _t_ (FJ) and spring temperature _t_ (ST). The posterior mean is displayed alongside their 95% credible intervals on a log scale. (b-d) Followed by their effects on recruitment rates predicted from an IPM.

**Table S1.** Mean parameter estimates and 95% credible intervals (CRIs) estimated from the IPM

| **Parameter** | **Mean** | **95% CRI** |
| --- | --- | --- |
| lambda[1] | 1.07 | (0.86,1.31) |
| lambda[2] | 1.42 | (1.18,1.72) |
| lambda[3] | 1.01 | (0.87,1.19) |
| lambda[4] | 1.17 | (1.01,1.37) |
| lambda[5] | 1.08 | (0.95,1.23) |
| lambda[6] | 0.93 | (0.82,1.05) |
| lambda[7] | 1.08 | (0.95,1.23) |
| lambda[8] | 1.08 | (0.94,1.23) |
| lambda[9] | 1.06 | (0.94,1.2) |
| lambda[10] | 1.03 | (0.91,1.15) |
| lambda[11] | 0.94 | (0.83,1.06) |
| lambda[12] | 0.97 | (0.85,1.11) |
| lambda[13] | 0.77 | (0.66,0.9) |
| lambda[14] | 1.30 | (1.1,1.53) |
| lambda[15] | 1.13 | (1,1.31) |
| lambda[16] | 0.84 | (0.74,0.96) |
| lambda[17] | 0.91 | (0.79,1.05) |
| lambda[18] | 0.87 | (0.74,1.02) |
| lambda[19] | 0.82 | (0.7,0.97) |
| lambda[20] | 0.98 | (0.83,1.14) |
| lambda[21] | 0.90 | (0.75,1.08) |
| lambda[22] | 0.95 | (0.78,1.13) |
| Ntot[1] | 75.55 | (61.08,90.81) |
| Ntot[2] | 79.89 | (67.01,93.43) |
| Ntot[3] | 113.06 | (96.67,129.85) |
| Ntot[4] | 114.24 | (100.02,129.05) |
| Ntot[5] | 133.25 | (116.7,151.21) |
| Ntot[6] | 143.50 | (127.8,160.2) |
| Ntot[7] | 132.65 | (117.9,148.25) |
| Ntot[8] | 143.44 | (127.4,161.3) |
| Ntot[9] | 154.41 | (137.5,172.11) |
| Ntot[10] | 163.43 | (146.2,180.85) |
| Ntot[11] | 167.22 | (149.6,185.7) |
| Ntot[12] | 156.11 | (139.85,173.8) |
| Ntot[13] | 151.17 | (134.65,168.4) |
| Ntot[14] | 115.78 | (100.1,133.5) |
| Ntot[15] | 149.87 | (132.9,167.4) |
| Ntot[16] | 169.58 | (151.1,189.31) |
| Ntot[17] | 142.61 | (126,160.1) |
| Ntot[18] | 129.66 | (114.25,146.56) |
| Ntot[19] | 112.25 | (96.61,128.5) |
| Ntot[20] | 91.65 | (79.59,105.2) |
| Ntot[21] | 89.41 | (76.26,103.4) |
| Ntot[22] | 80.12 | (68.79,93.32) |
| Ntot[23] | 75.82 | (62.97,90.17) |
| Recapture_Female | 0.78 | (0.76,0.8) |
| Recapture_ Male | 0.82 | (0.8,0.85) |

**Table S2.** The mean and 95% CRI of the posterior distribution of each regression R-squared.

|  | **Posterior mean R-squared** | **95% CRI** |
| --- | --- | --- |
| Female Survival~Disease | 0.24 | (0.09, 0.47) |
| Male Survival~Disease | 0.05 | (0.01, 0.29) |
| Female Survival~Density | 0.02 | (0.002, 0.22) |
| Male Survival~Density | 0.004 | (0.0001, 0.10) |
| Recruitment~Disease | 0.02 | (0.001, 0.46) |
| Recruitment~Density | 0.88 | (0.17, 0.95) |

1.

Macdonald, D.W., Newman, C., Buesching, C.D. & Nouvellet, P. (2010). Are badgers 'Under The Weather'? Direct and indirect impacts of climate variation on European badger (*Meles meles*) population dynamics. *Global Change Biology*, 16, 2913-2922.

2.

Pradel, R. (1996). Utilization of capture-mark-recapture for the study of recruitment and population growth rate. *Biometrics*, 52, 703-709.
